# Supplementary material for: Children’s Understanding of Informed Assents in Research Studies
Source: Healthcare (Basel). 2021 Jul 10;9(7):871. doi: 10.3390/healthcare9070871 (PMC8307200; doi:10.3390/healthcare9070871)
Supplement: Supplementary file 1 [file healthcare-09-00871-s001.zip › File 1_Ethics_Committee_Approval_HCVP.pdf]

Hospital Cruz Vermelha

*COMISSÃO DE INVESTIGAÇÃO, EDUCAÇÃO E ÉTICA*

**PARECER CIEE;02;2018,hl**

**PARA: Direcção Clínica do Hospital da Cruz Vermelha Portuguesa**
**Exmo Sr Dr Manuel Pedro Magalhães**
**Cc; Professora Doutora Teresa Magalhães**

**ASSUNTO:** Projecto de Investigação sob o tema ***"Impacto do Assentimento e do Consentimento numa População Vulnerável; O Olhar do Menor e do Tutor"***

No seguimento da reunião realizada pela Comissão a cinco de Julho de dois mil e dezoito, com o propósito de analisar o pedido supracitado, solicitado pela investigadora principal, Prof. Adjunta Hortense Maria Tavares Cotrim, Professora Adjunta convidada, e considerando os documentos entregues para avaliação e sua fundamentação, foi decidido por unanimidade dos elementos presentes, conforme registo em acta CIEE, ACTA02; 2018, dar parecer positivo ao plano de investigação, por o mesmo respeitar os princípios deontológicos e legais específicos para estas situações.

Lisboa, 5 de Julho de 2018

O Presidente Comissão Investigação, Educação e Ética

(Professor Doutor Aires de Sousa)

**Cópia para Investigadora Principal**
