# Supplementary material for: Children’s Understanding of Informed Assents in Research Studies
Source: Healthcare (Basel). 2021 Jul 10;9(7):871. doi: 10.3390/healthcare9070871 (PMC8307200; doi:10.3390/healthcare9070871)
Supplement: Supplementary file 1 [file healthcare-09-00871-s001.zip › File 2_Ethics_Committee_Approval_UCARDIO.pdf]

UCARDIO
Centro Clínico
Unidade Cardiovascular
Dr. Jorge Humberto Guardado
Director Clínico
Médico Cardiologista

PARECER
COMISSÃO DE ÉTICA PARA A SAÚDE DA SPAMEDIC,LDA., Nº1;2018

PARA: Direção Clínica da clínica UCARDIO
Dr. Jorge Humberto Guardado

ASSUNTO: Projeto de Investigação sob o tema:
"Impacto do Assentimento e do Consentimento numa População Vulnerável; O Olhar do Menor e do Tutor"

No Seguimento da reunião realizada pela comissão a sete de Julho de dois mil e dezoito, com o prepósito de analisar o pedido supracitado, solicitado pela investigadora principal, Prof. Adjunta Hortense Maria Tavares Cotrim, e considerando os documentes entregues para a análise, avaliação e argumentação, foi decidido por unanimidade dos elementos presentes votantes, conforme registo em acta da Comissão de Ética para a Saúde da Spamedic,Lda., Acta nº 01; 2018, dar parecer positivo ao plano de investigação, por o mesmo respeitar os princípios deontológicos e legais específicos para estas situações.

Torres Novas, 07 de Julho de 2018
O Presidente Comissão de Ética para a Saúde da Spamedic,Lda.,

(Dr. Luís Miguel Dória)

Cópia para Investigadora Principal
