# Supplementary material for: Children’s Understanding of Informed Assents in Research Studies
Source: Healthcare (Basel). 2021 Jul 10;9(7):871. doi: 10.3390/healthcare9070871 (PMC8307200; doi:10.3390/healthcare9070871)
Supplement: Supplementary file 1 [file healthcare-09-00871-s001.zip › File 3_Ethics Committee_approval_HPA.pdf]

Hospital Particular do Algarve

GRUPO HPA SAUDE

COMISSÃO DE ÉTICA PARA A SAÚDE DO HOSPITAL PARTICULAR DO ALGARVE

PARECER Nº 2/2018

Assunto: **Impacto do Assentimento e do consentimento numa população vulnerável: O Olhar do Menor e do Tutor**

Nos termos e para os efeitos do disposto na alínea a), do nº 1 do artº 9º da Lei nº 21/2014 de 16 de Abril, foi solicitado o parecer desta Comissão, tendo em vista a autorização para a realização do estudo acima identificado.

No âmbito das competência que lhe são conferidas pelo artº 6º do Decreto-Lei nº 97/95 de 10 de Maio e pelo do artº 16º da Lei nº 21/2014 de 16 de Abril, a CES-HPA analisou o processo que lhe foi apresentado para aquele efeito e avaliou todos os aspectos apontados no nº 6 do referido artº 16º.

1. Pertinência do estudo clínico e sua concepção:

De acordo com a informação prestada a esta Comissão a realização deste estudo torna-se pertinente uma vez que pretende demonstrar a importância dada pelo médico ao envolvimento dos menores na tomada de decisão relativa à sua autorização para execução de ecocardiografia de esforço

2. Avaliação dos benefícios e riscos previsíveis:

Foram referidos a esta comissão que o estudo não apresenta riscos previsíveis uma vez que se trata de um estudo descritivo simples e transversal no qual se vai utilizar a abordagem quantitativa.

3. O Protocolo, incluindo os planos de divulgação do estudo.

O estudo, pretende decorrer entre os 2018 e 2019.

Serão selecionados para o estudo todos os menores entre 10 e 18 anos que necessitem de ecocardiograma de esforço, com autonomia plena para o consentimento e ainda sobre a participação no estudo os pais/tutores.

4. Instrumento de colheita de dados

O instrumento de colheita de dados será o questionário a elaborar posteriormente e que será aplicado pelo médico e deverá ser sujeito a aprovação desta comissão de ética.

5. A aptidão do investigador principal e dos restantes membros da equipa.
Cada membro pesquisador está apto a exercer sua tarefa.
Sendo o Investigador Principal: Dra. Hortense Maria Cotrim sob a supervisão do Professor Doutor Carlos Cotrim e tendo como orientadores a Professora Doutora Cristina Granja e Professora Doutora Ana Sofia Carvalho atendendo ao tipo de estudo, parecem adequadas as qualificações dos profissionais responsáveis pelo estudo.

10. Procedimento de obtenção do consentimento informado incluindo as informações a prestar aos participantes.
O consentimento informado será explicado ao participante voluntário e/ou seu responsável legal durante a visita e antes da realização de qualquer procedimento do estudo.

Parece, pois, que neste protocolo de estudo e considerando os documentos entregues para avaliação e sua fundamentação são observados os compromissos éticos, o que permite que esta comissão profira um parecer favorável ao plano de investigação, e por o mesmo respeitar os princípios deontológicos e legais específicos para estas situações.

Portimão, 21 de Dezembro de 2018
