# Supplementary material for: Children’s Understanding of Informed Assents in Research Studies
Source: Healthcare (Basel). 2021 Jul 10;9(7):871. doi: 10.3390/healthcare9070871 (PMC8307200; doi:10.3390/healthcare9070871)
Supplement: Supplementary file 1 [file healthcare-09-00871-s001.zip › File 4_QuIC Part A and B English version.pdf]

QUALITY OF INFORMED ASSENT (QUIC), PART A

INSTRUCTIONS: Below you will find several statements about research studies. Thinking about your participation in this study, please read each statement carefully. Then tell us whether you agree with the statement, you disagree with the statement, or you are unsure about the statement by circling the appropriate response. Please respond to each statement as best you can. We are interested in your opinions.

A1. When I signed the consent form for the exercise echo, I knew I was agreeing to participate in a research study.	$Disagree_1$	$Unsure_2$	$Agree_3$
A2. The main reason for clinical studies is to improve the quality of care provided in the future.	$Disagree_1$	$Unsure_2$	$Agree_3$
A3. I was informed about the duration of my participation in this clinical study.	$Disagree_1$	$Unsure_2$	$Agree_3$
A4. All procedures in this study are meant to help clarify my possible health problem.	$Disagree_1$	$Unsure_2$	$Agree_3$
A5. One of the main goals of the researchers in this study is to create an assent model that can be applied to exercise echocardiography in children.	$Disagree_1$	$Unsure_2$	$Agree_3$
A 6. One of the main objectives of the researchers is to verify if the assent model used is suitable for the performance of exercise echocardiography.	$Disagree_1$	$Unsure_2$	$Agree_3$
A 7. One of the main goals of the researchers in this study is to make sure that I understand the information given to me about the possible risks of taking this exam.	$Disagree_1$	$Unsure_2$	$Agree_3$
A 8. Exercise echocardiography is considered the safest and least risky exam in the study of my possible health problem.	$Disagree_1$	$Unsure_2$	$Agree_3$
A 9. The need for performing an exercise echocardiography was decided by my doctor due to my possible health problem.	$Disagree_1$	$Unsure_2$	$Agree_3$
A 10. Compared to alternative complementary exams (angiotac, myocardial perfusion scintigraphy or catheterization), the exercise echocardiogram presents less risk or discomfort for my health.	$Disagree_1$	$Unsure_2$	$Agree_3$
A 11. There is a possibility that the exercise echocardiogram does not clarify the existing doubts about my possible health problem.	$Disagree_1$	$Unsure_2$	$Agree_3$
A 12. By participating in this clinical study, I am helping researchers acquire information that could benefit other children in the future.	$Disagree_1$	$Unsure_2$	$Agree_3$
13. The consent form I signed names the researchers, which I can contact if I have any questions or concerns about my participation in this study.	$Disagree_1$	$Unsure_2$	$Agree_3$
A 14. If I didn't want to participate in this clinical study, I could have refused to sign the consent form.	$Disagree_1$	$Unsure_2$	$Agree_3$
A 15. The doctor who performed the exercise echocardiogram explained to me, through clear and simple language, what the exam consisted of and its importance for my diagnosis and treatment.	$Disagree_1$	$Unsure_2$	$Agree_3$
A 16. The information that the doctor performing the exercise echocardiogram provided helped me calm down.	$Disagree_1$	$Unsure_2$	$Agree_3$
A 17. I felt more adult and responsible because the doctor asked me for permission to perform the exercise echocardiogram.	$Disagree_1$	$Unsure_2$	$Agree_3$

QUALITY OF INFORMED ASSENT (QuIC), PART B

<u>When you signed the assent form</u> to participate in this research study, how well did you understand the following aspects? *If you didn't understand the item at all, please circle **1**. If you understood it very well, please circle **5**. If you understand it somewhat, please circle a number between **1** and **5**.*

		I Didn't understand this at all				I understood This very well
B1.	Performing your exercise echocardiogram was used for an investigation.	1	2	3	4	5
B2.	Why are the researchers doing this study?	1	2	3	4	5
B3.	How long will you be participating in this study?	1	2	3	4	5
B4.	The procedures you will undergo.	1	2	3	4	5
B5.	Which of the procedures is experimental?	1	2	3	4	5
B6.	The possible risks and discomforts of participating in this study.	1	2	3	4	5
B7.	The possible benefits to you of participating in this study.	1	2	3	4	5
B8.	How your participation in this study may benefit future children.	1	2	3	4	5
B9.	The alternatives to your participation in this study (take the exam and not participate in the study).	1	2	3	4	5
B10.	The confidentiality of your participation in this study is guaranteed.	1	2	3	4	5
B11.	You do not have to pay any amount for participating in this study.	1	2	3	4	5
B12.	Whom you should contact if you have questions or concerns about the study.	1	2	3	4	5
B13.	The fact that your participation in this study is voluntary.	1	2	3	4	5
B14.	Overall, how well did you understand the study when you signed the consent form.	1	2	3	4	5
